# Synthesis, function, and regulation of sterol and nonsterol isoprenoids

**DOI:** 10.3389/fmolb.2022.1006822

**Published:** 2022-10-05

**Authors:** Rebecca Faulkner, Youngah Jo

**Affiliations:** Department of Molecular Genetics, University of Texas Southwestern Medical Center, Dallas, TX, United States

**Keywords:** mevalonate pathway, cholesterol, nonsterol isoprenoids, HMG CoA reductase, sterols

## Abstract

Cholesterol, the bulk end-product of the mevalonate pathway, is a key component of cellular membranes and lipoproteins that transport lipids throughout the body. It is also a precursor of steroid hormones, vitamin D, and bile acids. In addition to cholesterol, the mevalonate pathway yields a variety of nonsterol isoprenoids that are essential to cell survival. Flux through the mevalonate pathway is tightly controlled to ensure cells continuously synthesize nonsterol isoprenoids but avoid overproducing cholesterol and other sterols. Endoplasmic reticulum (ER)-localized 3-hydroxy-3-methylglutaryl coenzyme A (HMG CoA) reductase (HMGCR), the rate limiting enzyme in the mevalonate pathway, is the focus of a complex feedback regulatory system governed by sterol and nonsterol isoprenoids. This review highlights transcriptional and post-translational regulation of HMGCR. Transcriptional regulation of HMGCR is mediated by the Scap-SREBP pathway. Post-translational control is initiated by the intracellular accumulation of sterols, which causes HMGCR to become ubiquitinated and subjected to proteasome-mediated ER-associated degradation (ERAD). Sterols also cause a subfraction of HMGCR molecules to bind the vitamin K_2_ synthetic enzyme, UbiA prenyltransferase domain-containing protein-1 (UBIAD1). This binding inhibits ERAD of HMGCR, which allows cells to continuously synthesize nonsterol isoprenoids such as geranylgeranyl pyrophosphate (GGPP), even when sterols are abundant. Recent studies reveal that UBIAD1 is a GGPP sensor, dissociating from HMGCR when GGPP thresholds are met to allow maximal ERAD. Animal studies using genetically manipulated mice disclose the physiological significance of the HMGCR regulatory system and we describe how dysregulation of these pathways contributes to disease.

## 1 Introduction

**FIGURE 1:**
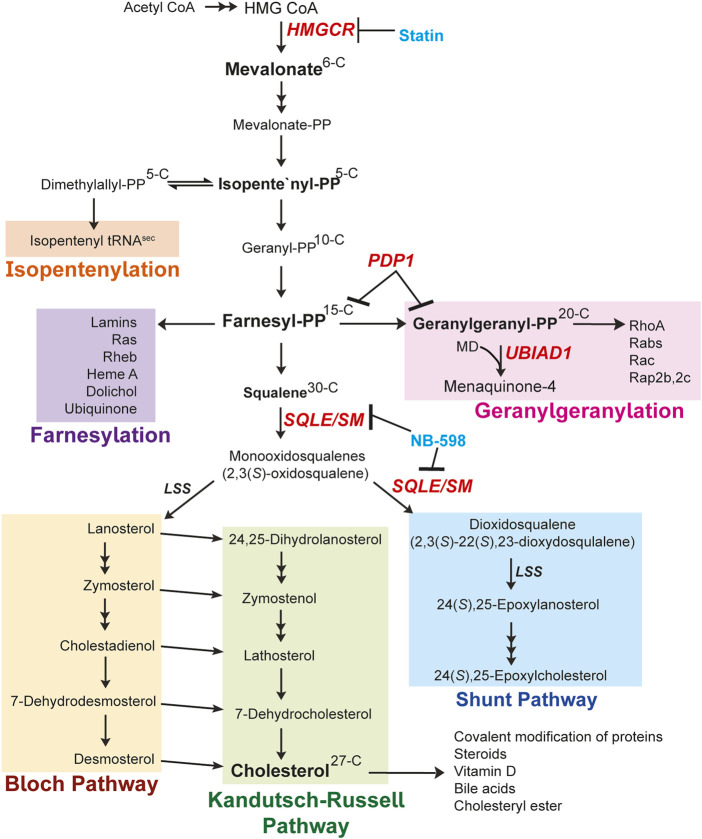
Mevalonate pathway (Brown and Goldstein, 1980; Borini Etichetti et al., 2020). Explanation of the pathway is described in the main text. Changes in carbon numbers are denoted by the right side of the molecules. The enzymes focused on in this review are indicated in red. The known inhibitors for enzymes are denoted in blue. Abbreviations: HMGCR, 3-hydroxy-3-methylglutaryl (HMG) Coenzyme A reductase; PP, pyrophosphate; MD, menadione; UBIAD1, UbiA prenyltransferase domain-containing protein-1; SQLE/SM, squalene epoxidase also called SM; LSS, lanosterol synthase; PDP1, type I polyisoprenoid diphosphate phosphatase 1 also called PPAPDC2.

Cholesterol is a hydrophobic lipid inserted in the phospholipid bilayer of biological membranes including the plasma membrane and other organelles such as the endoplasmic reticulum (ER), Golgi apparatus, mitochondria, nuclear membrane, etc. Cholesterol regulates the fluidity and rigidity of membranes and dynamically changes in response to environmental conditions. Cells obtain cholesterol by two mechanisms: 1) *de novo* synthesis from the 2-carbon precursor acetate through a series of more than 20 reactions that are collectively referred to as the mevalonate pathway; and 2) uptake of extracellular low-density lipoprotein (LDL) by the LDL receptor, which delivers LDL from the plasma membrane to lysosomes where free cholesterol is liberated. Cholesterol serves as a precursor for oxysterols, steroid hormones, vitamin D, and bile acids as shown in Figure 1. Cholesterol can become covalently attached to proteins such as Hedgehog, which plays a pivotal role in embryonic development (Porter et al., 1996; Mafi et al., 2021). The mevalonate pathway also produces a variety of sterol intermediates such as lanosterol, dihydrolanosterol (DHL), desmosterol, epoxycholesterol (EC), and dehydrocholesterols. The nonsterol branch of the pathway yields essential nonsterol isoprenoids including isopentenyl diphosphate/pyrophosphate (IPP), geranyl diphosphate/pyrophosphate (GPP), farnesyl diphosphate/pyrophosphate (FPP), and geranylgeranyl diphosphate/pyrophosphate (GGPP). From here diphosphate/pyrophosphate will be denoted as PP. These molecules are essential for normal cell function and are indispensable for several processes such as prenylation (farnesylation or geranylgeranylation) of small GTPases and synthesis of isopentenylated tRNA. Additionally, FPP and GGPP are utilized to synthesize other essential nonsterol isoprenoids such as ubiquinone-10 and heme (electron transport), dolichol (asparagine-linked glycoprotein synthesis) (Grabinska et al., 2016), and vitamin K_2_ (coagulation). Flux through the mevalonate pathway is tightly controlled by feedback loops that regulate the levels of key enzymes to ensure that cells continuously produce nonsterol isoprenoids but avoid over producing cholesterol and other sterols.

In this review we first describe the mevalonate pathway highlighting key enzymes subjected to feedback regulation by downstream metabolites (Figure 1). 3-Hydroxy-3-methylglutaryl coenzyme A (HMG CoA) reductase (HMGCR) is the rate limiting enzyme in the mevalonate pathway and as such, is subjected to tight feedback control through transcriptional and post-translational mechanisms. Transcriptional regulation of HMGCR is mediated by membrane-bound transcription factors called sterol-regulatory element-binding proteins that require the polytopic, cholesterol-regulated escort protein Scap for activation. The main focus centers on the post-translational regulation of HMGCR initiated by sterol and nonsterol isoprenoids which combine to accelerate ER-associated degradation (ERAD) of HMGCR. Feedback mechanisms by the isoprenoid branch are mediated by an HMGCR-associated protein called UbiA prenyltransferase domain-containing protein-1 (UBIAD1). Here we detail the current mechanistic understanding of the multivalent feedback pathways that act on HMGCR and UBIAD1 to maintain cellular homeostasis and suggest how dysregulation results in disease.

## 2 Overview of mevalonate pathway

### 2.1 Formation of isopentenyl pyrophosphate

**FIGURE 2:**
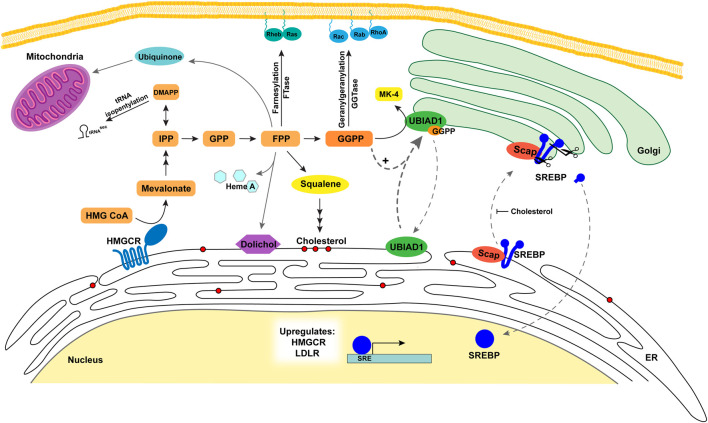
Metabolic roles and regulation of sterols and nonsterol isoprenoids in the cell. The Scap-SREBP complex is translocated to the Golgi where nuclear transcription factor SREBP-2 is released to increase transcription of HMGCR, LDLR, and other cholesterol biosynthetic enzymes. HMGCR expression is increased and catalyzes the conversion of HMG CoA to mevalonate increasing flux through the mevalonate pathway. The nonsterol metabolites (orange/yellow) serve as precursors for a variety of nonsterol isoprenoids that are vital for a number of cell processes. DMAPP is utilized for tRNA isopentenylation. FPP is converted to dolichol used in protein glycosylation in the ER, heme A, and ubiquinone for the mitochondria. FPP and GGPP are used for prenylation of proteins targeting them to membranes. GGPP is utilized with menadione for the formation of MK-4 by UBIAD1 in the Golgi. UBIAD1 cycles between the ER and the Golgi monitoring GGPP levels. As GGPP levels increase it binds UBIAD1 and is retained in the Golgi. FPP is also converted to squalene and is the first metabolite committed to the sterol synthesis branch of the pathway (red circles represent sterols). As cholesterol levels increase in ER membranes this inhibits Scap escorting SREBP to the Golgi for activation and oxysterols trigger Insig binding to HMGCR resulting in ERAD. Abbreviations: Scap, SREBP cleavage activating protein; SREBP, sterol regulatory element binding proteins; LDLR, low density lipoprotein receptor; HMGCR, 3-hydroxy-3-methylglutaryl (HMG) Coenzyme A reductase; IPP, isopentenyl diphosphate/pyrophosphate; FPP, farnesyl diphosphate/pyrophosphate; ER, endoplasmic reticulum; GGPP, geranylgeranyl diphosphate/pyrophosphate; MK-4, menaquinone-4; UBIAD1, UbiA prenyltransferase domain-containing protein-1.

Synthesis of cholesterol through the mevalonate pathway begins with the condensation of three acetyl CoA molecules forming HMG CoA by acetoacetyl CoA thiolase and HMG CoA synthase (Horton et al., 2002). HMG CoA is reduced to mevalonate by HMGCR, which resides in the ER membrane and is recognized as a rate-limiting step of the cholesterol biosynthetic pathway. Mevalonate is then subjected to successive phosphorylations by mevalonate kinase and phosphomevalonate kinase (PMK). Mevalonate pyrophosphate is decarboxylated by mevalonate pyrophosphate decarboxylase to produce the first isoprenoid, 5-carbon IPP, or its isomer dimethylallyl diphosphate/pyrophosphate (DMAPP). Additionally, the DMAPP molecule can become incorporated into tRNA, called **isopentenylation,** to produce isopentenylated selenocysteine tRNA^sec^. This 21st amino acid is incorporated into certain selenoproteins that participate in protein folding, degradation, calcium homeostasis and can become dysfunctional in neurodegenerative diseases (Gladyshev et al., 2016; Carlson et al., 2018; Fradejas-Villar et al., 2021). IPP and DMAPP are conjugated into the 10-carbon GPP and sequential additions of IPP are added to GPP to generate a 15-carbon unit called FPP. Both GPP and FPP are synthesized by the activity of FPP synthase (FPPS, farnesyl diphosphate synthase, FDPS). The FPP molecule serves as a major branch point in the pathway where it either is converted to squalene entering the sterol synthesis pathway or continues into the nonsterol isoprenoid branch Figure 1 and Figure 2).

### 2.2 Farnesyl pyrophosphate to nonsterol isoprenoids

In the nonsterol isoprenoid branch of the mevalonate pathway, GGPP synthase 1 (GGPPS1) extends FPP by adding IPP, generating the 20-carbon GGPP molecule (Kavanagh et al., 2006). Alternatively, two GPP (10-C) molecules are conjugated to form GGPP (20-C). The enzyme cis-prenyltransferase further extends FPP to produce dolichol, a glycosyl lipid carrier for glycosylation of proteins. It is also utilized for the formation of Heme A, an iron-chelating cofactor of cytochrome c oxidase involved in mitochondrial respiratory chain. Moreover, the trans-prenyltransferase utilizes FPP to produce poly-prenyl pyrophosphate that is subsequently transferred to 4-hydroxy-benzoate. This reaction constitutes the initial step in synthesis of ubiquinone-10 (CoQ10), which serves as an antioxidant by preventing mitochondrial oxidative stress. The prenyl groups of FPP (15-C) and GGPP (20-C) are covalently attached to cysteines by a thioester linkage in CAAX motifs on proteins by farnesyl transferase (**farnesylation**) and geranylgeranyl transferase I and II (protein **geranylgeranylation**), together referred to as **prenylation**. These modifications play pivotal regulatory roles by targeting proteins to membranes subsequently stimulating downstream signaling pathways. Finally, the enzyme UbiA prenyltransferase domain-containing protein-1 (UBIAD1) uses GGPP to prenylate menadione (MD, vitamin K_3_) released from dietary vitamin K_1_ to produce a subtype of vitamin K_2_ called menaquinone-4 (MK-4). Remarkably, UBIAD1 plays an essential role in the regulation of the mevalonate pathway and will be described later in this review.

### 2.3 Farnesyl pyrophosphate to cholesterol

The second biosynthetic outcome of FPP is the synthesis of cholesterol. Two FPP molecules are condensed to form squalene by squalene synthase (also called farnesyl diphosphate farnesyltransferase 1, FDFT1). Squalene is the first metabolite committed to the synthesis of sterols and is converted to 2,3(*S*)-oxidosqualene (monooxidosqualene) by squalene monooxygenase (SM) also called squalene epoxidase (SQLE). SM (SQLE) is recognized as a key regulatory enzyme of the sterol portion of the pathway and is subjected to post-translational regulation by cholesterol (Gill et al., 2011). Monooxidosqualene is cyclized by lanosterol synthase (cyclase) to generate lanosterol, the first sterol metabolite produced exhibiting the characteristic four ring steroid nucleus. Lanosterol has two fates: 1) it continues through the Bloch pathway to produce cholesterol or 2) it is hydoxylated by 3β-hydroxysterol Δ24-reductase (DHCR24/Seladin-1) forming 24,25-dihydrolanosterol (DHL) that enters the Kandutsch-Russell pathway to form cholesterol (Kandutsch and Russell, 1960; Bloch, 1965). The flux through both pathways has been recently measured *in vitro* and *in vivo*. These studies revealed that many different cells and tissue types generate cholesterol through both pathways. However, steroidogenic tissues such as testes and adrenal glands utilize exclusively the canonical Bloch pathway and the study demonstrated that regulation of cholesterol biosynthesis was through the Bloch pathway (Mitsche et al., 2015).

### 2.4 Shunt pathway

Monooxidosqualene is alternatively converted to 2,3(*S*)-22(*S*),23-dioxidosqualene which is then cyclized by lanosterol synthase (cyclase) to 24(*S*),25-epoxylanosterol as the first metabolite in the Shunt Pathway. Through several steps 24(*S*),25-epoxylanosterol is metabolized to 24(*S*),25-epoxycholesterol by CYP7B1. Studies indicate that epoxycholesterols play a vital role in the development of the brain as well as other tissues. Reports show 24(*S*),25-epoxycholesterol plays a variety of roles during development and is abundant in mouse ventral midbrain. It activates the liver X receptor (LXR) in the ventral midbrain during development to induce stem cell differentiation into dopaminergic neurons (Broccoli and Caiazzo, 2013; Theofilopoulos et al., 2019). Findings demonstrate 24(*S*),25-epoxycholesterol inhibits IL-6 production and degranulation of bone marrow-derived murine mast cells that express LXRβ (Nunomura et al., 2010). It inhibits the conversion of desmosterol to cholesterol by DHCR24/Seladin-1 in CHO-7 and SRD-1 cells (Zerenturk et al., 2012). This inhibition suggests 24(*S*),25-epoxycholesterol triggers feedback mechanisms to regulate the mevalonate pathway and more understanding is needed.

## 3 Mechanisms of sterol sensing by Scap-SREBP

Owing to its insolubility in aqueous solution, the overaccumulation of cholesterol must be avoided because it can form crystals that trigger cell death. Thus, a stringent feedback regulatory system is employed to maintain cholesterol homeostasis, while at the same time allowing production of essential nonsterol isoprenoids. Exogenous LDL-cholesterol is removed from the extracellular milieu by the low-density lipoprotein receptor (LDLR) through receptor-mediated endocytosis. The LDLR delivers LDL-cholesterol to lysosomes where most of the liberated cholesterol is incorporated into the plasma membrane. Once levels of cholesterol reach a certain threshold in the plasma membrane, LDL-derived cholesterol is delivered to the ER membrane through a mechanism that involves phosphatidylserine and GRAMD1/Aster proteins (Trinh et al., 2022). The precise mechanisms for this intracellular trafficking of cholesterol remains to be determined. The delivery of cholesterol to ER membrane reduces *de novo* cholesterol synthesis by inhibiting transactivation of genes encoding cholesterol biosynthetic enzymes and the LDLR.

Transcriptional regulation of the mevalonate pathway is mediated by a family of transcription factors called SREBPs (sterol regulatory element binding proteins). SREBPs are unusual transcription factors because they are synthesized as precursors bound to membranes of the ER with an N-terminal transcription factor domain, two transmembrane helices separated by a short loop, and a large cytosolic C-terminal regulatory domain (Figure 2). SREBP precursors are associated with the escort protein Scap. The N-terminal domain of Scap contains eight membrane spanning helices and a C-terminal domain that interacts with SREBPs through its regulatory C-terminal domain (Nohturfft et al., 1998). When cells are deprived of cholesterol, Scap escorts SREBPs to the Golgi (Radhakrishnan et al., 2008). In the Golgi, the N-terminal DNA-binding domain of SREBP-2 is released from the membrane (Brown et al., 2018) by sequential cleavages catalyzed by a serine protease called Site-1 protease (S1P) and the metalloprotease Site-2 protease (S2P). The mature N-terminal domain of SREBP translocates to the nucleus and enhances transcription of target genes required for the synthesis and uptake of cholesterol, including HMGCR and LDLR genes respectively. Conversely, when cholesterol accumulates and reaches 5 mol% of total ER lipids, Scap binds to cholesterol, which triggers a conformational change in the protein that allows it to bind to ER membrane proteins called Insig-1 or Insig-2 (Yabe et al., 2002; Yang et al., 2002). This binding blocks incorporation of Scap-SREBP complexes into COPII-coated vesicles, resulting in their sequestration in the ER (Espenshade et al., 2002). ER sequestration of Scap-SREBP prevents proteolytic activation of SREBPs, blunting cholesterol synthesis and uptake (Yang et al., 2002). Human Insig-1 and Insig-2 contain six transmembrane domains and exhibit 59% identity. Although they play redundant roles in the regulation of Scap-SREBP, they are differentially regulated in livers of mice. Insig-1 is a target gene of SREBPs, while expression of Insig-2 is enhanced under fasting or hypoxic conditions (Engelking et al., 2004; Hwang et al., 2017).

Scap contains within its membrane domain a region compromising transmembrane helices 2-6 called the sterol sensing domain (SSD) that mediates the sterol-induced binding to Insig (Sever et al., 2003a; Feramisco et al., 2004). Importantly, introduction of point mutations within the SSD of Scap abolishes its binding to Insigs, permitting continued transport to the Golgi and proteolytic activation of SREBPs in the presence of sterols. Indeed, recent cryogenic electron microscopy (cryo-EM) studies reveal that the Scap-Insig complex is maintained by a hydrophobic interface comprised of transmembrane domains 2, 4, and 5 of Scap and 1, 3, and 4 of Insig (Kober et al., 2021; Yan et al., 2021). At least five other proteins that are related to cholesterol metabolism contain an SSD within their membrane domains (Nohturfft et al., 1998; Sever et al., 2003b). These proteins include HMGCR, Niemann Pick Type C 1 (NPC1) and NPC1-Like-1 (NPC1L1), which mediate cellular uptake of LDL-derived and dietary cholesterol, respectively (Davies and Ioannou, 2000; Kwon et al., 2009; Kwon et al., 2011), and Patched and Dispatched that are modulated by cholesterol-modified Hedgehog (Zhong and Wang, 2022).

## 4 Sterol feedback regulation through HMGCR

**FIGURE 3:**
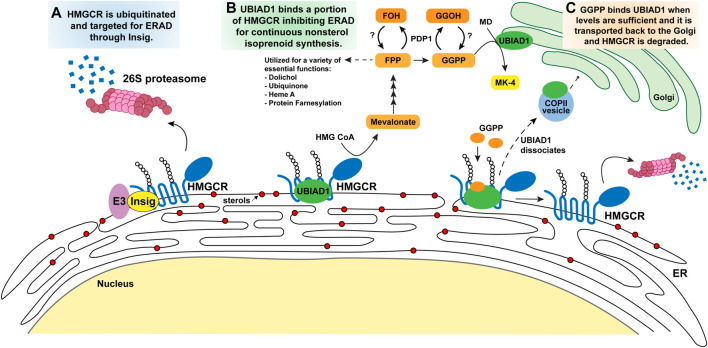
Model for the regulatory mechanisms of HMGCR and UBIAD1 by the mevalonate pathway. Accumulation of intracellular sterols (DHL, lanosterol, and certain oxysterols all represented as red circles) in the ER membrane **(A)** trigger Insig binding to the membrane domain of HMGCR. Insig-associated E3 ligases ubiquitinate HMGCR on cytosolic lysine residues (K89, K248) marking the protein for degradation by 26S proteosomes and subsequently diminishing mevalonate production and flux through the pathway. **(B)** Following Insig-mediated ubiquitination of HMGCR, a subset of HMGCR molecules is bound by UBIAD1 thereby inhibiting its ERAD. This allows the cell to continue to synthesize a low level of mevalonate for nonsterol isoprenoids necessary for cellular processes illustrated in Figure 1. GGPP is the last nonsterol isoprenoid produced and serves as a feedback molecule. **(C)** As GGPP levels increase to sufficient levels it binds UBIAD1 that is complexed with HMGCR, triggering UBIAD1 dissociation from HMGCR, and UBIAD1 is transported from the ER to the Golgi in COPII vesicles. HMGCR released from UBIAD1 is subjected to ERAD. Abbreviations: HMGCR, 3-hydroxy-3-methylglutaryl (HMG) Coenzyme A reductase; UBIAD1, UbiA prenyltransferase domain-containing protein-1; DHL, 24,25-dihydrolanosterol; ER, endoplasmic reticulum; GGPP, geranylgeranyl pyrophosphate; PDP1, type I polyisoprenoid diphosphate phosphatase 1 or phosphatidic acid phosphatase type 2 domain containing 2.

Since cloning of the cDNA encoding HMGCR in 1984 (Chin et al., 1984), extensive studies have sought to understand the complex regulatory mechanisms that govern the level and activity of the enzyme. This regulatory system is multivalent and involves transcriptional, translational, and post-translational mechanisms (Brown and Goldstein, 1980; Nakanishi et al., 1988). Transcriptional regulation of HMGCR is mediated by SREBPs, whose activation is described in Section 3. Nonsterol isoprenoids mediate the translational regulation of HMGCR; however, the mechanism for the response is completely unknown, but may involve the 5’ untranslated region of HMGCR mRNA (Nakanishi et al., 1988). Here we will focus on the post-translational mechanism of the HMGCR regulatory system that is mediated by sterols (lanosterol, DHL, and oxysterols) and the nonsterol isoprenoid GGPP (Figure 3).

### 4.1 Post-translational regulation of HMGCR

The HMGCR cDNA encodes a protein that consists of 887 to 888 amino acids, which can be divided into an N-terminal domain (388 amino acids) with eight transmembrane helices integrated in ER membranes and a C-terminal domain projects into the cytosol and exerts the enzyme’s catalytic activity (Liscum et al., 1985; Jo and Debose-Boyd, 2010). In the 1970’s and 1980’s studies demonstrated that the activity of HMGCR can be regulated by reversible phosphorylation that is controlled by intracellular levels of cholesterol (Beg et al., 1978; Mitropoulos et al., 1980; Von Gunten and Sinensky, 1989; Smythe et al., 1998). This modification is regulated by AMP activated protein kinase on the residue serine 871 of rat HMGCR and quickly responds to changes in cellular energy levels (Gillespie and Hardie, 1992; Sato et al., 1993). The phosphorylation system has been extensively studied in fission yeast with the homologues of human Insig-1 and HMGCR termed, Ins1 and Hmg1p (Burg et al., 2008). The Hmg1p can be phosphorylated on a conserved residues of serine 1024 corresponding to serine 871/872 of rat/human HMGCR respectively, and a nonconserved residue threonine 1028, which are responsible for rapidly responding to physiological changes in nutrient levels. Here, Ins1-mediated phosphorylation of Hmg1p is regulated by the yeast MAP kinase called Sty1/Spc1. The functions of Insig-1 in the yeast and mammalian systems are likely similar as a negative regulator of Hmg1p in fission yeast and mammalian HMGCR, but the mechanisms are somewhat different in the two different systems.

Here, we focus on a well-studied mechanism for post-translational control of HMGCR by ER-associated degradation (ERAD). Early studies disclosed that when cells are treated with sterols, the degradation of HMGCR is accelerated more than 5-fold. Two observations demonstrate that the membrane domain of HMGCR is crucial for this ERAD (Roitelman and Simoni, 1992). First, deletion of the membrane domain of HMGCR yielded a catalytically active C-terminal domain whose ERAD was not accelerated by sterols (Gil et al., 1985). The second line of evidence was provided by studies from Bob Simoni and co-workers who showed that a chimeric protein consisting of β-galactosidase fused to the membrane domain of HMGCR exhibited sterol-accelerated ERAD similar to that of wild-type HMGCR (Skalnik et al., 1988; Roitelman et al., 1992). These findings form the basis for the conclusion that the membrane domain is the target of post-translational regulation of HMGCR.

The complexity of HMGCR ERAD was first revealed by studies that employed compactin, a competitive inhibitor of HMGCR isolated from fungi and the first statin drug (Endo et al., 1976). When cells were incubated with compactin and lipoprotein deficient serum, HMGCR protein dramatically accumulated (>200 fold), owing to depletion of mevalonate metabolites that mediates feedback regulation of the enzyme (Brown et al., 1978). Supplying cells with LDL or various oxysterols accelerated ERAD of HMGCR; however, the complete reversal of compactin-mediated effects required the further addition of mevalonate (Roitelman and Simoni, 1992). These results formed the basis for the conclusion that mevalonate-derived sterol and nonsterol isoprenoids combine to accelerate HMGCR ERAD, reducing its half-life more than 10-fold (Von Gunten and Sinensky, 1989). Subsequent reports indicated that sterols stimulated ubiquitination of HMGCR, thereby marking the protein for proteasome-mediated ERAD from membranes (Inoue et al., 1991; Ravid et al., 2000).

### 4.2 HMGCR regulation by Insig-mediated sterol-accelerated ER-associated degradation

Uncovering the roles of Insigs in the sterol-regulated ER retention of the Scap-SREBP complex (Yabe et al., 2002) provided key insight into mechanisms for sterol-accelerated ERAD of HMGCR. Transmembrane helices 2–6 of both Scap and HMGCR contain a SSD necessary for Insig binding through a conserved tetrapeptide sequence YIYF; mutations in this sequence abolish Insig binding and render the HMGCR resistant to degradation. HMGCR and Scap appear to bind to the same site on Insigs as overexpression of the SSD of Scap titrates Insig from HMGCR preventing its degradation (Sever et al., 2003a). Conversely, under low sterol conditions, Insigs do not bind SCAP or HMGCR and the unbound Insig quickly undergoes ERAD with a half-life of approximately 1 h.

Extensive studies identified the E3 ubiquitin ligase, gp78, responsible for Insig-1 ubiquitination and degradation by the 26S proteasome (Figure 3A) under steady state condition (Song et al., 2005a). In contrast, when cellular sterols are high, Insig-1 associates with sterols, which leads to stabilizing conformational changes in Insig-1 where it is recruited to HMGCR bringing with it gp78. Other ER resident E3 ligases, TRC8 and RNF145, have additionally been shown to ubiquitinate HMGCR in an Insig dependent manner (Jo et al., 2011; Menzies et al., 2018). In knock down or knock out experiments targeting ligases gp78, TRC8 and RNF145, ERAD of HMGCR was abolished. The ubiquitination sites on the HMGCR membrane domain were identified as two cytosolically exposed lysine residues that are adjacent to transmembrane helices 3 and 7, respectively. When residues K89 and K248 are mutated to arginine by site-directed mutagenesis, the membrane domain of HMGCR is no longer ubiquitinated and degradation of the protein is blocked (Sever et al., 2003a). However, HMGCR (K89R, K248R) still interacts with Insig proteins further demonstrating the role these sites play in the ubiquitination of HMGCR. This overall regulatory system is conserved in *Drosophila* and yeast suggesting that the feedback loop is fundamental in cell survival (Nguyen et al., 2009; Faulkner et al., 2013).

Two mouse models were generated to explore the physiological significance of HMGCR ERAD. In the first model, mice were generated that express in the liver a transgene encoding the membrane domain of HMGCR [HMGCR (TM1-8)] (Hwang et al., 2016). Expression of this transgene was driven by the human apolipoprotein E (apoE) promoter which is widely used to generate a liver-specific expression of a gene of interest. Thus, any change in expression of HMGCR (TM1-8) could be attributed to modulation of the protein’s ERAD. Feeding transgenic mice with a diet supplemented with cholesterol led to acceleration of HMGCR (TM1-8) ERAD. Conversely, the protein accumulated when the mice were challenged with a diet containing the statin lovastatin. The second mouse model constituted mice harboring knock-in mutations that changed lysines 89 and 248 to arginine. These mice were designated *Hmgcr* ^*Ki/Ki*^(Hwang et al., 2016). HMGCR accumulated in livers of *Hmgcr* ^*Ki/Ki*^; this accumulation occurred despite the overaccumulation of cholesterol that suppressed activation of SREBPs and reduction of HMGCR mRNA. When *Hmgcr* ^*Ki/Ki*^ mice were fed a cholesterol diet, HMGCR protein was resistant to ERAD compared to that in wild type counterparts. Finally, the statin induced accumulation of hepatic HMGCR is blunted 5-fold in these mice when compared to wild-type, indicating that ERAD contributes to the statin induced accumulation of HMGCR and is a potential novel drug target to lower cholesterol synthesis.

Since cholesterol itself does not regulate HMGCR ERAD *in vitro*, many studies have sought to identify the sterol product responsible for these feedback mechanisms. Models focus on the influence of hydroxylated sterols, of which roles were demonstrated in early studies (Brown and Goldstein, 1974; Kandutsch and Chen, 1975). These reports indicate that oxygenated sterols, especially 25-hydroxycholesterol and 7-ketocholesterol, can suppress the activity of HMGCR substantially. However, for complete suppression of HMGCR activity, oxygenated sterols also require a nonsterol product provided through the addition of mevalonate. An additional study demonstrates that the 24,25-dihydrolanosterol regulates the activity of HMGCR, but still needs mevalonate for complete degradation of the HMGCR (Song et al., 2005b). Together, this data concludes that both sterol and nonsterol products are required for complete suppression of HMGCR activity. The mechanism of lanosterol or 24,25-dihydrolanosterol in ubiquitination of HMGCR is not clear, and it is unknown if these sterols directly associate with HMGCR.

## 5 Nonsterol isoprenoid regulation through HMGCR ERAD

### 5.1 Nonsterol requirement

The identity of the nonsterol isoprenoid that combines with sterols to maximally accelerate HMGCR ERAD remained a mystery until 2003 (Sever et al., 2003a). This study showed that the nonsterol requirement for HMGCR ERAD could be fulfilled by the addition of geranylgeraniol (GGOH), the alcohol derivative of GGPP. It is believed that the treated GGOH is converted to a biologically active form, GGPP, in cells by an unknown kinase. Our recent work indirectly supports this idea by showing that when the GGPP dephosphorylating enzyme is transiently overexpressed *in vitro* the effect of GGOH on HMGCR ERAD is blunted (Elsabrouty et al., 2021) as described in Section 5.3. The effect of GGOH was remarkably specific in that farnesol (FOH), the alcohol derivative of FPP, did not combine with sterols to accelerate HMGCR ERAD (Sever et al., 2003a). Although GGOH triggered ERAD of HMGCR, the isoprene did not enhance its sterol-induced binding to Insigs or ubiquitination. Instead, GGOH modulated one of the two sequential post-ubiquitination steps in HMGCR ERAD. The first post-ubiquitination step involves the extraction of ubiquitinated HMGCR across ER membranes by valosin-containing protein (VCP)/p97, which belongs to the ATPases associated with diverse cellular activities (AAA) superfamily of ATPases. Biochemical evidence indicates that GGOH enhances VCP/p97-mediated extraction of HMGCR (Elsabrouty et al., 2013). In the second post-ubiquitination step, extracted HMGCR is dislodged from the ER membrane into the cytosol by the 19S regulatory particle of the proteasome (19S RP), which contains six AAA-ATPases. Following cytosolic dislocation, HMGCR is delivered into the core of the 20S proteasome for degradation.

### 5.2 Geranylgeranyl pyrophosphate sensing through UbiA prenyltransferase domain-containing protein 1

Our group sought to identify the target protein of GGOH and understand its role in regulating HMGCR ERAD (Figures 3B,C). Through a proximity biotinylation assay of associated HMGCR proteins, a protein called UbiA prenyltransferase domain-containing protein 1 (UBIAD1) was identified by mass spectrometry (Schumacher et al., 2015). UBIAD1, also referred to as transitional epithelial response protein-1 (TERE1), was initially discovered for its role in the synthesis of the vitamin K_2_ subtype menaquinonen-4 (MK-4) (Nakagawa et al., 2010). UBIAD1 catalyzes transfer of a geranylgeranyl group from GGPP to menadione (MD or vitamin K_3_) that is, derived from phylloquinone (PK or vitamin K_1_), obtained through dietary green leafy vegetables and meats.

We discovered that sterols stimulate the binding of UBIAD1 to HMGCR through a reaction that required the presence of Insigs (Schumacher et al., 2015). Contrastingly, GGOH caused dissociation of the HMGCR-UBIAD1 complex. Our group characterized the role UBIAD1 plays in the stabilization of HMGCR and continues to investigate how it regulates the nonsterol isoprenoid pathway. Overexpressing UBIAD1 in a cell system stabilizes the HMGCR protein in the ER, even under excess amounts of sterols administered in conjunction with compactin (Schumacher et al., 2015). GGOH-induced dissociation of this complex was specific inasmuch as FOH failed to block the interaction. The stabilization of UBIAD1-HMGCR is dissociated by the addition of GGPP or GGOH, leading to maximal HMGCR degradation, and the UBIAD1 enzyme is transported from the ER to Golgi. The dissociation is specific to GGPP, and FPP or FOH did not have any effect, suggesting that UBIAD1 is a sensor of GGPP for inhibiting HMGCR degradation. When UBIAD1 expression was silenced by RNA interference-mediated knockdown or CRISPR/Cas9-mediated knockout, HMGCR ERAD is no longer stimulated by GGPP further confirming its regulatory role in HMGCR ERAD relieved the requirement of GGOH for maximal ERAD of HMGCR. In these UBIAD1 deficient cells sterols alone stimulate HMGCR ubiquitination and degradation even in the presence of compactin, indicating the UBIAD1 inhibits the reaction. During our studies, we found that UBIAD1 unexpectedly localized to the medial-trans Golgi when cells were cultured under isoprenoid-replete conditions (Schumacher et al., 2016). However, when the cells were deprived of nonsterol isoprenoids by compactin, UBIAD1 rapidly translocates from the Golgi to the ER. Golgi localization of UBIAD1 was restored by GGOH, but not by FOH. In addition, blocking protein export from the ER results in the accumulation of UBIAD1 in the ER, even when cells are replete with isoprenoids. Taken together, these findings indicate that UBIAD1 constitutively cycles between membranes of the Golgi and ER. Upon depletion of GGOH (or GGPP), UBIAD1 becomes trapped in the ER to block HMGCR ERAD, allowing synthesis of mevalonate that becomes incorporated into nonsterol isoprenoids.

Clinical studies identified missense mutations in human UBIAD1 that cause the autosomal-dominant eye disease Schnyder Corneal Dystrophy (SCD) (Weiss et al., 2007). Patients harboring SCD-associated mutations in UBIAD1 exhibit progressive opacification of the cornea owing to abnormally high levels of cholesterol/lipids in the tissue. This opacification results in vision loss and many patients require corneal transplants (Weiss et al., 2008; Al-Ghadeer et al., 2011; Du et al., 2011; Tao et al., 2012; Xie and Li, 2021). Structures of bacterial UbiA prenyltransferases have been reported; the active sites of UbiA prenyltransferases are well-conserved across species. SCD-associated mutations can be mapped around the GGPP binding site of UbiA prenyltransferases, suggesting they may interfere with GGPP sensing. Indeed, all SCD-associated variants of UBIAD1 are sequestered in the ER and refractory to GGOH-induced transport to the Golgi. Moreover, they block ERAD of HMGCR in a dominant-negative fashion.

Point mutations that change asparagine-102 in UBIAD1 to serine (N102S) is one of the most frequent mutations in SCD families. To examine the physiological role of UBIAD1 in HMGCR ERAD, we generated mice designated *Ubiad1* ^*Ki/Ki*^ that harbor knock-in mutations that change asparagine-100 to serine (N100S) (Jo et al., 2019). This mutation corresponds to the N102S mutation in human UBIAD1. Similar to results with *Hmgcr* ^*Ki/Ki*^ mice (Section 4.2), HMGCR protein accumulated in liver and other tissues of *Ubiad1* ^*Ki/Ki*^ mice and the protein was resistant to ERAD stimulated by cholesterol feeding. As a result of this resistance, the animals overproduced cholesterol as well as nonsterol isoprenoids including GGOH and ubiquinone-10. The statin-induced accumulation of HMGCR was also blunted in livers of *Ubiad1* ^*Ki/Ki*^ mice. Subcellular localization studies revealed that lovastatin caused UBIAD1 to translocate from the Golgi to ER in livers of wild type mice, whereas the protein remained in hepatic ER membranes of *Ubiad1* ^*Ki/Ki*^ regardless of absence or presence of lovastatin. Aged *Ubiad1* ^*Ki/Ki*^ mice (>1 year) exhibited signs of corneal opacification, sterol accumulation, and down-regulation of SREBP processing (Jiang et al., 2019; Jo et al., 2019). Importantly, studies using mouse embryonic fibroblasts (MEFs) from *Ubiad1* ^*Ki/Ki*^ provided direct evidence that UBIAD1 inhibits a post-ubiquitination step in HMGCR ERAD. Despite the marked accumulation of HMGCR in *Ubiad1* ^*Ki/Ki*^ (compared to wild type control), the protein continued to become ubiquitinated in the presence of sterols. *In vitro* studies together with cryo-electron microscopy structures of the HMGCR-UBIAD1(N102S) complex revealed HMGCR TM 7 interacts with TMs 2-4 of UBIAD1, and complex formation is disrupted by mutagenesis of specific residues. Our group continues to study the precise mechanisms through which UBIAD1 inhibits HMGCR ERAD, and the implications of these interactions have on the statin-induced accumulation of HMGCR (Chen et al., 2022).

A group characterized the role of UBIAD1 in MK-4 biosynthesis in cells distinct from PK which is supplied from diet, and from other forms of vitamin K_2_ provided by gut bacteria (Nakagawa et al., 2010). This group also reported the germ line knockout of UBIAD1 in mice, which demonstrated embryonic lethal and failed to rescue by the feeding of MK-4 through pregnancy, concluding the important role of MK-4 in development (Nakagawa et al., 2014). However, we observed that our UBIAD1 deficient cell lines displayed accelerated HMGCR ERAD resulting in reduced products of the mevalonate pathway. This gave us the idea to use our previously discussed *Hmgcr* ^*Ki/Ki*^ mice that are resistant to degradation and have an overproduction of HMGCR in their tissues, to rescue the embryonic lethality of UBIAD1 deficiency. Using this approach, we successfully generated UBIAD1 knockout mice in *Hmgcr* ^*Ki/Ki*^ mice (*Ubiad1* ^*KO*^ *; Hmgcr* ^*KI*^). As reported previously, *Ubiad1* ^*KO*^ mouse line expressing normal HMGCR did not give birth to any UBIAD1 knockout homozygotes, while *Ubiad1* ^*KO*^ *; Hmgcr* ^*KI*^ produced homozygotes UBIAD1 knockout mice at the expected Mendelian ratio. This indicated that the nondegradable form of HMGCR^KI^ can rescue the embryonic lethality of *Ubiad1* ^*KO*^ mouse line (Jo et al., 2020). We characterized the *Ubiad1* ^*KO*^ *; Hmgcr* ^*KI*^ mice which displayed a lack of MK-4 synthesis in all tested tissues and physiological defects in bone growth and muscle regeneration, demonstrating potential roles of UBIAD1 and MK-4 in these tissues and in-depth studies are underway. Experiments were attempted to rescue the phenotype by feeding a MK-4 supplemented diet, but it was not successful. We suspect this is because all forms of vitamin K undergo the cleavage of side chains and are converted to MD in enterocytes, making UBIAD1 vital to fill this role (Ellis et al., 2022).

### 5.3 Dephosphorylation of geranylgeranyl pyrophosphate by polyisoprenoid diphosphate phosphatase 1

Our work demonstrates that cells monitor nonsterol isoprenoid needs by using UBIAD1 as a sensor to screen levels of GGPP and thereby regulating ERAD of HMGCR (Elsabrouty et al., 2021). These studies show GGPP is the most effective isoprenoid for regulating the UBIAD1-HMGCR complex dissociation, indicating the significant physiological roles of GGPP in regulating downstream functions such as vitamin K synthesis or modification of various proteins as shown in Figure 2. Our group sought to understand what regulates GGPP levels in the cell. Another group previously identified and characterized an enzyme called type I polyisoprenoid diphosphate phosphatase 1 (PDP1 or PPAPDC2) (Miriyala et al., 2010). PDP1 is localized in the ER membrane and plays a role in hydrolysis of polyisoprenoid diphosphates FPP and GGPP preferentially over various phospholipids and sphingolipids (Figures 1, 3C). Our studies showed overexpression of PDP1 led to depletion of polyisoprenoid diphosphates FPP and GGPP, causing decreases in protein prenylation in targets such as Rho family GTPases, resulting in defective cytoskeletal organization and eventually cell death. The role of PDP1 in dephosphorylation of FPP and GGPP has a significant effect on the reduced prenylation of small GTPases and ERAD of HMGCR through UBIAD1 (Elsabrouty et al., 2021). The knock down of PDP1 by RNA interference led to an increase in cellular GGPP levels, which facilitated the dissociation of UBIAD1 and HMGCR accelerating ERAD of HMGCR. This accumulation of GGPP resulting from PDP1 knockdown is marked by an increase in MK-4 synthesis. This increased cellular GGPP also affects small GTPase geranylgeranylation, which leads to translocation of small GTPases to target membrane organelles, and to accelerated HMGCR ERAD making it a potential future drug target. However, the endogenous kinase involved in the phosphorylation of the alcohol forms of isoprenoids FOH or GGOH is not yet identified.

## 6 Regulation through squalene monooxygenase/epoxidase

Synthesis of one cholesterol molecule from acetyl CoA through the mevalonate pathway requires 11 oxygen molecules (Summons et al., 2006; Brown and Galea, 2010). Squalene (30-C) is synthesized by squalene synthase (FDFT1) from two FPP (15-C) molecules, and oxidized by squalene monooxygenase (SM, squalene epoxidase, SQLE) to produce 2,3-epoxysqualene, the first step of oxygenation in sterol biosynthesis. This conversion by squalene monooxygenase is considered the second regulatory step in the cholesterol synthetic pathway (Gill et al., 2011; Yoshioka et al., 2020). This regulation was first observed when cholesterol treated cells displayed a marked accumulation of squalene, and accelerated degradation of SM/SQLE by proteasomal activity through its N-terminal degron (Gill et al., 2011). This group further demonstrated that the N-terminal region of SM is responsible for direct interaction with cholesterol rather than squalene leading to cholesterol-induced degradation of SM/SQLE through MARCH6 (Membrane-associated Ring Finger protein 6, TEB4, RNF176) E3 ubiquitin ligase (Zelcer et al., 2014). There are striking differences between the degradation of SM/SQLE and HMGCR. SM/SQLE degradation is not likely mediated by Insig proteins or regulated by 24,25-dihydrolanosterol or oxysterols, but rather by cholesterol. SM/SQLE activity and protein expression is unchanged by statins, and a SM/SQLE inhibitor and been identified, NB-598. Several studies have suggested that SM/SQLE is increased in a pan-cancer genome wide screening, which indicates genes related to survival of cancer under hypoxia conditions (Haider et al., 2016). In colorectal cancers displaying cholesterol accumulation, SM/SQLE expression was decreased, and correlated with accelerated cancer progression and metastasis (Jun et al., 2021). A recent study in advanced prostate cancer showed SM/SQLE was increased and its regulator microRNA-205 (miRNA-205) was lowered. Here, the progression of cancer was able to be down regulated by the overexpression of miRNA-205 or by the treatment of SM/SQLE inhibitors (Kalogirou et al., 2021). Some cancers such as lymphoma showed elevated levels of squalene and displayed cholesterol auxotrophy. The lymphoma displayed down regulation of SM/SQLE which led to increased squalene and altered lipid profiles resulting in ferroptosis inhibition and cancer cell survival (Kalogirou et al., 2021).

## 7 Regulation of mevalonate pathway through hypoxia

Oxygen deprivation (hypoxia) is sensed by mevalonate pathway intermediates, and results in increased HMGCR ERAD. The multi-step conversion of squalene to cholesterol consumes 11 oxygen molecules. During hypoxic conditions the demethylation of lanosterol and its close metabolite 24,25-dihydrolanosterol is slowed and accumulates in the cell (Nguyen et al., 2007). Additionally, Insig proteins are increased by transcriptional activation through hypoxia-inducible factor (HIF)-1α. Insigs, particularly Insig-2 in human fibroblast cells and mouse liver, is actively transcribed and synthesized by HIF-1α. Increased Insig proteins together with 24,25-dihydrolanosterol accumulation triggers accelerated HMGCR ERAD. These findings were further confirmed in mouse models (Hwang et al., 2017). However, it is not known whether SM/SQLE is also regulated by hypoxia. Since hypoxia is tightly associated with the tumor environment, further investigation is needed for cell or cancer type-specific regulation of HMGCR, Insigs or lanosterol species through HIF-1α for clinical therapeutic applications.

## 8 Traveling between the organelles

The development of the electron microscopy ultrastructure of cellular organelles redefined our understanding of cellular transport (Palade, 1975). Well characterized intracellular transport systems include clathrin-coated vesicles for the endocytic pathway from the plasma membrane to endosomes, COPII vesicles from the ER to Golgi anterograde, and COPI from Golgi to ER retrograde transport (Lee et al., 2004; Mehrani and Stagg, 2022). Cells utilize ER to Golgi transport to regulate sterol and nonsterol biosynthesis by two mechanisms, the recycling of Scap-SREBP and UBIAD1 which is mediated by COPII and COPI protein complexes. Regulation of Scap-SREBP transport is controlled by cholesterol and 25-hydroxycholesterol induced Insig binding which blocks the Scap-SREBP transport to COPII vesicles on the ER by inhibition of Sar1-dependent coat protein binding Sec23/24 (Espenshade et al., 2002; Sun et al., 2005). Under low sterol conditions, the COPII binding site on amino acid residue Y640 in loop 6 of Scap is exposed facilitating the movement of the Scap-SREBP complex to the Golgi. Sec23, one of the coat proteins of COPII components, is delayed being turned over under sterol depleted condition, to facilitate the budding of the cargo (Sun et al., 2007; Brown and Goldstein, 2009; Zhang et al., 2013). Another study also suggests that cholesterol binds to loop1 of Scap, causing the conformational change preventing the exit of Scap by precluding the COPII binding (Motamed et al., 2011). The other regulatory protein that cycles between the ER-Golgi is UBIAD1. It most likely utilizes COPII and COPI vesicles to cycle between the two organelles as evidenced in a simple budding assay from isolated microsomes where GGPP can induce the budding of UBIAD1 in small vesicles (Aridor et al., 1998; Schumacher et al., 2016; Elsabrouty et al., 2021). Our studies show UBIAD1 resides in the Golgi under high GGPP conditions and is translocated to the ER when GGPP levels are low stabilizing a portion of HMGCR for continued isoprenoid synthesis. Therefore, our model is that UBIAD1 cycles between the ER and Golgi sampling GGPP levels using anterograde and retrograde transport systems. However, the exact molecular mechanism has not yet been dissected.

## 9 Conclusions and future scope

This overview of the regulatory mechanisms that govern the mevalonate pathway, combines early discoveries made with recent studies from our group. In summary, cells work to maintain flux through the sterol and nonsterol isoprenoid branches of the pathway by influencing HMGCR ERAD through distinct mechanisms. Sterol metabolites produced, such as the oxysterol 25-HC, interact with Insig proteins which in turn bind HMGCR and target it for ERAD. However, methylated sterols such as DHL and lanosterol trigger HMGCR ERAD, but do not bind Insigs or activate SREBP processing. Additionally, the bisphosphonate esters SR-12813 and apomine mimic methylated sterols in triggering HMGCR ERAD without binding Insigs, and current efforts are underway to determine if they act through direct interactions with HMGCR or through an associated protein. Understanding the mechanisms for sterol sensing will shed light for future drug development.

When sterol levels are abundant in the ER membranes, cells maintain a low level of HMGCR for isoprenoid synthesis. This portion of HMGCR is protected from ERAD by the binding of UBIAD1 which is regulated by GGPP levels. Accumulation of GGPP disrupts UBIAD1 binding to HMGCR, and UBIAD1 is translocated to the Golgi until GGPP levels change. The unbound HMGCR protein is then subjected to ERAD. UBIAD1 cycles between the Golgi and the ER monitoring GGPP levels and regulating HMGCR ERAD to maintain the necessary mevalonate needed for isoprenoid synthesis. A newly discovered point of regulation by our group is the conversion of GGPP to GGOH by PDP1 which could potentially be targeted to modulate GGPP levels, and thereby influence HMGCR stabilization by UBIAD1.

A hallmark of many cancer types is increased flux through the mevalonate pathway to ensure continuous growth and survival. This is achieved through numerous mechanisms including dysregulating key enzymes and altering transcriptional tumor suppressors to support cell proliferation and tumor metastasis (Gobel et al., 2020; Juarez and Fruman, 2021). Although statins, competitive inhibitors of HMGCR, have been widely prescribed for decades to treat hypercholesterolemia, these drugs have been recently repurposed for anti-cancer therapy. These studies utilizing various cancer cell lines and animal models have revealed statin treatment can trigger tumor-specific apoptosis (Gupta et al., 2013; Tsubaki et al., 2016; Alizadeh et al., 2017; Deng et al., 2019; Gobel et al., 2019; Kuzyk et al., 2020). Some patients in clinical trials have exhibited promising results while others experience statin resistance, illustrating the need for other drug targets and alternative approaches (Clendening et al., 2010; Kimbung et al., 2016). It is highly controversial whether UBIAD1 plays a role in anti-cancer effect or cancer progression. UBIAD1 has been known as a tumor suppressor for urological cancer, castrate-resistant prostate cancer and renal cell carcinoma (Fredericks et al., 2013). Also, the role of UBIAD1 in synthesis of non-mitochondrial CoQ10 was beneficial for the melanoma cells since it prevented lipid peroxidation and cell death (Arslanbaeva et al., 2022). It is speculated that the UBIAD1 product, MK-4, has a beneficial role in the protection of diverse types of cancer, however an in-depth investigation is needed in these areas.

## Glossary

25-HC: 25-hydroxycholesterol
DHCR24: 3β-hydroxysterol Δ24-reductase also called Seladin-1
DHL: 24,25-dihydrolanosterol
DMAPP: dimethylallyl diphosphate/pyrophosphate
EC: epoxycholesterol
ERAD: Endoplasmic reticulum associated degradation
FDFT1: farnesyl diphosphate farnesyltransferase 1: fanesyl diphosphate farnesyl transferase 1
FPP: farnesyl diphosphate/pyrophosphate
FDPS/FPPS: farnesyl diphosphate synthase
FDFT1: farnesyl diphosphate farnesyltransferase 1: fanesyl diphosphate farnesyl transferase 1
GGPP: geranylgeranyl diphosphate/pyrophosphate
GGPPS1: GGPP synthase 1
GPP: geranyl diphosphate/pyrophosphate
HIF: hypoxia-inducible factor
HMGCR: 3-hydroxy-3-methylglutaryl (HMG) Coenzyme A reductase
Insig: insulin-induced gene
IPP: isopentenyl diphosphate/pyrophosphate
LDL: low density lipoprotein
LDLR: low density lipoprotein receptor
LSS: lanosterol synthase
LXR: liver X receptor
MD: menadione, vitamin K_3_
MK-4: menaquinone-4, one of the vitamin K_2_
NPC: Niemann Pick Type C
NPC1L1: NPC1-Like-1
PDP1 or PPAPDC2: type I polyisoprenoid diphosphate phosphatase 1 or phosphatidic acid phosphatase type 2 domain containing 2
PMK: phosphomevalonate kinase
Scap: SREBP cleavage activating protein
SM: squalene monooxygenase also called SQLE
SREBP: sterol regulatory element binding proteins
SSD: sterol sensing domain
SQLE: squalene epoxidase also called SM
UBIAD1: UbiA prenyltransferase domain-containing protein-1

## References

[B1] Al-Ghadeer H. Mohamed J. Y. Khan A. O. (2011). Schnyder corneal dystrophy in a Saudi arabian family with heterozygous UBIAD1 mutation (p.L121F). Middle East Afr. J. Ophthalmol. 18 (1), 61–64. 10.4103/0974-9233.75890 21572737 PMC3085155

[B2] Alizadeh J. Zeki A. A. Mirzaei N. Tewary S. Rezaei Moghadam A. Glogowska A. (2017). Mevalonate cascade inhibition by simvastatin induces the intrinsic apoptosis pathway via depletion of isoprenoids in tumor cells. Sci. Rep. 7, 44841. 10.1038/srep44841 28344327 PMC5366866

[B3] Aridor M. Weissman J. Bannykh S. Nuoffer C. Balch W. E. (1998). Cargo selection by the COPII budding machinery during export from the ER. J. Cell Biol. 141 (1), 61–70. 10.1083/jcb.141.1.61 9531548 PMC2132735

[B4] Arslanbaeva L. Tosi G. Ravazzolo M. Simonato M. Tucci F. A. Pece S. (2022). UBIAD1 and CoQ10 protect melanoma cells from lipid peroxidation-mediated cell death. Redox Biol. 51, 102272. 10.1016/j.redox.2022.102272 35255427 PMC8902599

[B5] Beg Z. H. Stonik J. A. Brewer H. B. Jr. (1978). 3-Hydroxy-3-methylglutaryl coenzyme A reductase: Regulation of enzymatic activity by phosphorylation and dephosphorylation. Proc. Natl. Acad. Sci. U. S. A. 75 (8), 3678–3682. 10.1073/pnas.75.8.3678 278983 PMC392849

[B6] Bloch K. (1965). The biological synthesis of cholesterol. Science 150 (3692), 19–28. 10.1126/science.150.3692.19 5319508

[B7] Borini Etichetti C. M. Arel Zalazar E. Cocordano N. Girardini J. (2020). Beyond the mevalonate pathway: Control of post-prenylation processing by mutant p53. Front. Oncol. 10, 595034. 10.3389/fonc.2020.595034 33224889 PMC7674641

[B8] Broccoli V. Caiazzo M. (2013). Nuclear receptors: Oxysterols detour to neurodevelopment. Nat. Chem. Biol. 9 (2), 70–71. 10.1038/nchembio.1165 23334545

[B9] Brown A. J. Galea A. M. (2010). Cholesterol as an evolutionary response to living with oxygen. Evolution 64 (7), 2179–2183. 10.1111/j.1558-5646.2010.01011.x 20394667

[B10] Brown M. S. Faust J. R. Goldstein J. L. Kaneko I. Endo A. (1978). Induction of 3-hydroxy-3-methylglutaryl coenzyme A reductase activity in human fibroblasts incubated with compactin (ML-236B), a competitive inhibitor of the reductase. J. Biol. Chem. 253 (4), 1121–1128. 10.1016/s0021-9258(17)38120-6 624722

[B11] Brown M. S. Goldstein J. L. (2009). Cholesterol feedback: From schoenheimer's bottle to scap's MELADL. J. Lipid Res. 50, S15–S27. 10.1194/jlr.R800054-JLR200 18974038 PMC2674699

[B12] Brown M. S. Goldstein J. L. (1980). Multivalent feedback regulation of HMG CoA reductase, a control mechanism coordinating isoprenoid synthesis and cell growth. J. Lipid Res. 21 (5), 505–517. 10.1016/s0022-2275(20)42221-7 6995544

[B13] Brown M. S. Goldstein J. L. (1974). Suppression of 3-hydroxy-3-methylglutaryl coenzyme A reductase activity and inhibition of growth of human fibroblasts by 7-ketocholesterol. J. Biol. Chem. 249 (22), 7306–7314. 10.1016/s0021-9258(19)42106-6 4436312

[B14] Brown M. S. Radhakrishnan A. Goldstein J. L. (2018). Retrospective on cholesterol homeostasis: The central role of Scap. Annu. Rev. Biochem. 87, 783–807. 10.1146/annurev-biochem-062917-011852 28841344 PMC5828883

[B15] Burg J. S. Powell D. W. Chai R. Hughes A. L. Link A. J. Espenshade P. J. (2008). Insig regulates HMG-CoA reductase by controlling enzyme phosphorylation in fission yeast. Cell Metab. 8 (6), 522–531. 10.1016/j.cmet.2008.09.004 19041767 PMC2646361

[B16] Carlson B. A. Lee B. J. Tsuji P. A. Copeland P. R. Schweizer U. Gladyshev V. N. (2018). Selenocysteine tRNA([Ser]Sec), the central component of selenoprotein biosynthesis: Isolation, identification, modification, and sequencing. Methods Mol. Biol. 1661, 43–60. 10.1007/978-1-4939-7258-6_4 28917036 PMC5836751

[B111] Chen H. Qi X. Faulkner R. A. Schumacher M. M. Donnelly L. M. DeBose-Boyd R. A. (2022). Regulated degradation of HMG CoA reductase requires conformational changes in sterol-sensing domain. Nat. Commun. 13 (1), 4273. 10.1038/s41467-022-32025-5 35879350 PMC9314443

[B17] Chin D. J. Gil G. Russell D. W. Liscum L. Luskey K. L. Basu S. K. (1984). Nucleotide sequence of 3-hydroxy-3-methyl-glutaryl coenzyme A reductase, a glycoprotein of endoplasmic reticulum. Nature 308 (5960), 613–617. 10.1038/308613a0 6546784

[B18] Clendening J. W. Pandyra A. Boutros P. C. El Ghamrasni S. Khosravi F. Trentin G. A. (2010). Dysregulation of the mevalonate pathway promotes transformation. Proc. Natl. Acad. Sci. U. S. A. 107 (34), 15051–15056. 10.1073/pnas.0910258107 20696928 PMC2930553

[B19] Davies J. P. Ioannou Y. A. (2000). Topological analysis of Niemann-Pick C1 protein reveals that the membrane orientation of the putative sterol-sensing domain is identical to those of 3-hydroxy-3-methylglutaryl-CoA reductase and sterol regulatory element binding protein cleavage-activating protein. J. Biol. Chem. 275 (32), 24367–24374. 10.1074/jbc.M002184200 10821832

[B20] Deng H. Y. Lan X. Zheng X. Zha P. Zhou J. Wang R. L. (2019). The association between statin use and survival of esophageal cancer patients: A systematic review and meta-analysis. Med. Baltim. 98 (29), e16480. 10.1097/MD.0000000000016480 PMC6709309 31335710

[B21] Du C. Li Y. Dai L. Gong L. (2011). A mutation in the UBIAD1 gene in a Han Chinese family with Schnyder corneal dystrophy. Mol. Vis. 17, 2685–2692. 22065921 PMC3209473

[B22] Ellis J. L. Fu X. Karl J. P. Hernandez C. J. Mason J. B. DeBose-Boyd R. A. (2022). Multiple dietary vitamin K forms are converted to tissue menaquinone-4 in mice. J. Nutr. 152 (4), 981–993. 10.1093/jn/nxab332 34550377 PMC8971004

[B23] Elsabrouty R. Jo Y. Dinh T. T. DeBose-Boyd R. A. (2013). Sterol-induced dislocation of 3-hydroxy-3-methylglutaryl coenzyme A reductase from membranes of permeabilized cells. Mol. Biol. Cell 24 (21), 3300–3308. 10.1091/mbc.E13-03-0157 24025715 PMC3814148

[B24] Elsabrouty R. Jo Y. Hwang S. Jun D. J. DeBose-Boyd R. A. (2021). Type 1 polyisoprenoid diphosphate phosphatase modulates geranylgeranyl-mediated control of HMG CoA reductase and UBIAD1. Elife 10, e64688. 10.7554/eLife.64688 34842525 PMC8641950

[B25] Endo A. Kuroda M. Tanzawa K. (1976). Competitive inhibition of 3-hydroxy-3-methylglutaryl coenzyme A reductase by ML-236A and ML-236B fungal metabolites, having hypocholesterolemic activity. FEBS Lett. 72 (2), 323–326. 10.1016/0014-5793(76)80996-9 16386050

[B26] Engelking L. J. Kuriyama H. Hammer R. E. Horton J. D. Brown M. S. Goldstein J. L. (2004). Overexpression of Insig-1 in the livers of transgenic mice inhibits SREBP processing and reduces insulin-stimulated lipogenesis. J. Clin. Invest. 113 (8), 1168–1175. 10.1172/JCI20978 15085196 PMC385408

[B27] Espenshade P. J. Li W. P. Yabe D. (2002). Sterols block binding of COPII proteins to SCAP, thereby controlling SCAP sorting in ER. Proc. Natl. Acad. Sci. U. S. A. 99 (18), 11694–11699. 10.1073/pnas.182412799 12193656 PMC129331

[B28] Faulkner R. A. Nguyen A. D. Jo Y. DeBose-Boyd R. A. (2013). Lipid-regulated degradation of HMG-CoA reductase and Insig-1 through distinct mechanisms in insect cells. J. Lipid Res. 54 (4), 1011–1022. 10.1194/jlr.M033639 23403031 PMC3653402

[B29] Feramisco J. D. Goldstein J. L. Brown M. S. (2004). Membrane topology of human insig-1, a protein regulator of lipid synthesis. J. Biol. Chem. 279 (9), 8487–8496. 10.1074/jbc.M312623200 14660594

[B30] Fradejas-Villar N. Bohleber S. Zhao W. Reuter U. Kotter A. Helm M. (2021). The effect of tRNA([Ser]Sec) isopentenylation on selenoprotein expression. Int. J. Mol. Sci. 22 (21), 11454. 10.3390/ijms222111454 34768885 PMC8583801

[B31] Fredericks W. J. Sepulveda J. Lai P. Tomaszewski J. E. Lin M. F. McGarvey T. (2013). The tumor suppressor TERE1 (UBIAD1) prenyltransferase regulates the elevated cholesterol phenotype in castration resistant prostate cancer by controlling a program of ligand dependent SXR target genes. Oncotarget 4 (7), 1075–1092. 10.18632/oncotarget.1103 23919967 PMC3759667

[B32] Gil G. Faust J. R. Chin D. J. Goldstein J. L. Brown M. S. (1985). Membrane-bound domain of HMG CoA reductase is required for sterol-enhanced degradation of the enzyme. Cell 41 (1), 249–258. 10.1016/0092-8674(85)90078-9 3995584

[B33] Gill S. Stevenson J. Kristiana I. Brown A. J. (2011). Cholesterol-dependent degradation of squalene monooxygenase, a control point in cholesterol synthesis beyond HMG-CoA reductase. Cell Metab. 13 (3), 260–273. 10.1016/j.cmet.2011.01.015 21356516

[B34] Gillespie J. G. Hardie D. G. (1992). Phosphorylation and inactivation of HMG-CoA reductase at the AMP-activated protein kinase site in response to fructose treatment of isolated rat hepatocytes. FEBS Lett. 306 (1), 59–62. 10.1016/0014-5793(92)80837-7 1628744

[B35] Gladyshev V. N. Arner E. S. Berry M. J. Brigelius-Flohe R. Bruford E. A. Burk R. F. (2016). Selenoprotein gene nomenclature. J. Biol. Chem. 291 (46), 24036–24040. 10.1074/jbc.M116.756155 27645994 PMC5104929

[B36] Gobel A. Breining D. Rauner M. Hofbauer L. C. Rachner T. D. (2019). Induction of 3-hydroxy-3-methylglutaryl-CoA reductase mediates statin resistance in breast cancer cells. Cell Death Dis. 10 (2), 91. 10.1038/s41419-019-1322-x 30692522 PMC6349912

[B37] Gobel A. Rauner M. Hofbauer L. C. Rachner T. D. (2020). Cholesterol and beyond - the role of the mevalonate pathway in cancer biology. Biochim. Biophys. Acta. Rev. Cancer 1873 (2), 188351. 10.1016/j.bbcan.2020.188351 32007596

[B38] Grabinska K. A. Park E. J. Sessa W. C. (2016). cis-Prenyltransferase: New insights into protein glycosylation, rubber synthesis, and human diseases. J. Biol. Chem. 291 (35), 18582–18590. 10.1074/jbc.R116.739490 27402831 PMC5000101

[B39] Gupta S. C. Sung B. Prasad S. Webb L. J. Aggarwal B. B. (2013). Cancer drug discovery by repurposing: Teaching new tricks to old dogs. Trends Pharmacol. Sci. 34 (9), 508–517. 10.1016/j.tips.2013.06.005 23928289

[B40] Haider S. McIntyre A. van Stiphout R. G. P. M. Winchester L. M. Wigfield S. Harris A. L. (2016). Genomic alterations underlie a pan-cancer metabolic shift associated with tumour hypoxia. Genome Biol. 17 (1), 140. 10.1186/s13059-016-0999-8 27358048 PMC4926297

[B41] Horton J. D. Goldstein J. L. Brown M. S. (2002). SREBPs: Activators of the complete program of cholesterol and fatty acid synthesis in the liver. J. Clin. Invest. 109 (9), 1125–1131. 10.1172/JCI15593 11994399 PMC150968

[B42] Hwang S. Hartman I. Z. Calhoun L. N. Garland K. Young G. A. Mitsche M. A. (2016). Contribution of accelerated degradation to feedback regulation of 3-Hydroxy-3-methylglutaryl coenzyme A reductase and cholesterol metabolism in the liver. J. Biol. Chem. 291 (26), 13479–13494. 10.1074/jbc.M116.728469 27129778 PMC4919435

[B43] Hwang S. Nguyen A. D. Jo Y. Engelking L. J. Brugarolas J. DeBose-Boyd R. A. (2017). Hypoxia-inducible factor 1α activates insulin-induced gene 2 (Insig-2) transcription for degradation of 3-hydroxy-3-methylglutaryl (HMG)-CoA reductase in the liver. J. Biol. Chem. 292 (22), 9382–9393. 10.1074/jbc.M117.788562 28416613 PMC5454117

[B44] Inoue S. Bar-Nun S. Roitelman J. Simoni R. D. (1991). Inhibition of degradation of 3-hydroxy-3-methylglutaryl-coenzyme A reductase *in vivo* by cysteine protease inhibitors. J. Biol. Chem. 266 (20), 13311–13317. 10.1016/s0021-9258(18)98840-x 1906466

[B45] Jiang S. Y. Tang J. J. Xiao X. Qi W. Wu S. Jiang C. (2019). Schnyder corneal dystrophy-associated UBIAD1 mutations cause corneal cholesterol accumulation by stabilizing HMG-CoA reductase. PLoS Genet. 15 (7), e1008289. 10.1371/journal.pgen.1008289 31323021 PMC6668851

[B46] Jo Y. Debose-Boyd R. A. (2010). Control of cholesterol synthesis through regulated ER-associated degradation of HMG CoA reductase. Crit. Rev. Biochem. Mol. Biol. 45 (3), 185–198. 10.3109/10409238.2010.485605 20482385 PMC2937355

[B47] Jo Y. Hamilton J. S. Hwang S. Garland K. Smith G. A. Su S. (2019). Schnyder corneal dystrophy-associated UBIAD1 inhibits ER-associated degradation of HMG CoA reductase in mice. Elife 8, e44396. 10.7554/eLife.44396 30785396 PMC6402834

[B48] Jo Y. Kim S. S. Garland K. Fuentes I. DiCarlo L. M. Ellis J. L. (2020). Enhanced ER-associated degradation of HMG CoA reductase causes embryonic lethality associated with Ubiad1 deficiency. Elife 9, e54841. 10.7554/eLife.54841 32118581 PMC7069719

[B49] Jo Y. Lee P. C. W. Sguigna P. V. DeBose-Boyd R. A. (2011). Sterol-induced degradation of HMG CoA reductase depends on interplay of two Insigs and two ubiquitin ligases, gp78 and Trc8. Proc. Natl. Acad. Sci. U. S. A. 108 (51), 20503–20508. 10.1073/pnas.1112831108 22143767 PMC3251157

[B50] Juarez D. Fruman D. A. (2021). Targeting the mevalonate pathway in cancer. Trends Cancer 7 (6), 525–540. 10.1016/j.trecan.2020.11.008 33358111 PMC8137523

[B51] Jun S. Y. Brown A. J. Chua N. K. Yoon J. Y. Lee J. J. Yang J. O. (2021). Reduction of squalene epoxidase by cholesterol accumulation accelerates colorectal cancer progression and metastasis. Gastroenterology 160 (4), 1194–1207. 10.1053/j.gastro.2020.09.009 32946903

[B52] Kalogirou C. Linxweiler J. Schmucker P. Snaebjornsson M. T. Schmitz W. Wach S. (2021). MiR-205-driven downregulation of cholesterol biosynthesis through SQLE-inhibition identifies therapeutic vulnerability in aggressive prostate cancer. Nat. Commun. 12 (1), 5066. 10.1038/s41467-021-25325-9 34417456 PMC8379214

[B53] Kandutsch A. A. Chen H. W. (1975). Regulation of sterol synthesis in cultured cells by oxygenated derivatives of cholesterol. J. Cell. Physiol. 85 (1), 415–424. 10.1002/jcp.1040850408 164478

[B54] Kandutsch A. A. Russell A. E. (1960). Preputial gland tumor sterols. J. Biol. Chem. 235, 2256–2261. 10.1016/s0021-9258(18)64608-3 14404284

[B55] Kavanagh K. L. Dunford J. E. Bunkoczi G. Russell R. G. G. Oppermann U. (2006). The crystal structure of human geranylgeranyl pyrophosphate synthase reveals a novel hexameric arrangement and inhibitory product binding*. J. Biol. Chem. 281 (31), 22004–22012. 10.1074/jbc.M602603200 16698791

[B56] Kimbung S. Lettiero B. Feldt M. Bosch A. Borgquist S. (2016). High expression of cholesterol biosynthesis genes is associated with resistance to statin treatment and inferior survival in breast cancer. Oncotarget 7 (37), 59640–59651. 10.18632/oncotarget.10746 27458152 PMC5312337

[B57] Kober D. L. Radhakrishnan A. Goldstein J. L. Brown M. S. Clark L. D. Bai X. C. (2021). Scap structures highlight key role for rotation of intertwined luminal loops in cholesterol sensing. Cell 184 (14), 3689–3701. 10.1016/j.cell.2021.05.019 34139175 PMC8277531

[B58] Kuzyk C. L. Anderson C. C. Roede J. R. (2020). Simvastatin induces delayed apoptosis through disruption of glycolysis and mitochondrial impairment in neuroblastoma cells. Clin. Transl. Sci. 13 (3), 563–572. 10.1111/cts.12740 31917509 PMC7214657

[B59] Kwon H. J. Abi-Mosleh L. Wang M. L. Deisenhofer J. Goldstein J. L. Brown M. S. (2009). Structure of N-terminal domain of NPC1 reveals distinct subdomains for binding and transfer of cholesterol. Cell 137 (7), 1213–1224. 10.1016/j.cell.2009.03.049 19563754 PMC2739658

[B60] Kwon H. J. Palnitkar M. Deisenhofer J. (2011). The structure of the NPC1L1 N-terminal domain in a closed conformation. PLoS One 6 (4), e18722. 10.1371/journal.pone.0018722 21525977 PMC3078110

[B61] Lee M. C. Miller E. A. Goldberg J. Orci L. Schekman R. (2004). Bi-directional protein transport between the ER and Golgi. Annu. Rev. Cell Dev. Biol. 20, 87–123. 10.1146/annurev.cellbio.20.010403.105307 15473836

[B62] Liscum L. Finer-Moore J. Stroud R. M. Luskey K. L. Brown M. S. Goldstein J. L. (1985). Domain structure of 3-hydroxy-3-methylglutaryl coenzyme A reductase, a glycoprotein of the endoplasmic reticulum. J. Biol. Chem. 260 (1), 522–530. 10.1016/s0021-9258(18)89764-2 3965461

[B63] Mafi A. Purohit R. Vielmas E. Lauinger A. R. Lam B. Cheng Y. S. (2021). Hedgehog proteins create a dynamic cholesterol interface. PLoS One 16 (2), e0246814. 10.1371/journal.pone.0246814 33630857 PMC7906309

[B64] Mehrani A. Stagg S. M. (2022). Probing intracellular vesicle trafficking and membrane remodelling by cryo-EM. J. Struct. Biol. 214 (1), 107836. 10.1016/j.jsb.2022.107836 35101600 PMC8923612

[B65] Menzies S. A. Volkmar N. Van den Boomen D. J. Timms R. T. Dickson A. S. Nathan J. A. (2018). The sterol-responsive RNF145 E3 ubiquitin ligase mediates the degradation of HMG-CoA reductase together with gp78 and Hrd1. Elife 7, e40009. 10.7554/eLife.40009 30543180 PMC6292692

[B66] Miriyala S. Subramanian T. Panchatcharam M. Ren H. McDermott M. I. Sunkara M. (2010). Functional characterization of the atypical integral membrane lipid phosphatase PDP1/PPAPDC2 identifies a pathway for interconversion of isoprenols and isoprenoid phosphates in mammalian cells. J. Biol. Chem. 285 (18), 13918–13929. 10.1074/jbc.M109.083931 20110354 PMC2859554

[B67] Mitropoulos K. A. Knight B. L. Reeves B. E. (1980). 3-hydroxy-3-methylglutaryl-coenzyme A reductase A comparison of the modulation *in vitro* by phosphorylation and dephosphorylation to modulation of enzyme activity by feeding cholesterol- or cholestryamine-supplemented diets. Biochem. J. 185 (2), 435–441. 10.1042/bj1850435 6249255 PMC1161370

[B68] Mitsche M. A. McDonald J. G. Hobbs H. H. Cohen J. C. (2015). Flux analysis of cholesterol biosynthesis *in vivo* reveals multiple tissue and cell-type specific pathways. Elife 4, e07999. 10.7554/eLife.07999 26114596 PMC4501332

[B69] Motamed M. Zhang Y. Wang M. L. Seemann J. Kwon H. J. Goldstein J. L. (2011). Identification of luminal Loop 1 of Scap protein as the sterol sensor that maintains cholesterol homeostasis. J. Biol. Chem. 286 (20), 18002–18012. 10.1074/jbc.M111.238311 21454655 PMC3093874

[B70] Nakagawa K. Hirota Y. Sawada N. Yuge N. Watanabe M. Uchino Y. (2010). Identification of UBIAD1 as a novel human menaquinone-4 biosynthetic enzyme. Nature 468 (7320), 117–121. 10.1038/nature09464 20953171

[B71] Nakagawa K. Sawada N. Hirota Y. Uchino Y. Suhara Y. Hasegawa T. (2014). Vitamin K2 biosynthetic enzyme, UBIAD1 is essential for embryonic development of mice. PLoS One 9 (8), e104078. 10.1371/journal.pone.0104078 25127365 PMC4134213

[B72] Nakanishi M. Goldstein J. L. Brown M. S. (1988). Multivalent control of 3-hydroxy-3-methylglutaryl coenzyme A reductase. Mevalonate-derived product inhibits translation of mRNA and accelerates degradation of enzyme. J. Biol. Chem. 263 (18), 8929–8937. 10.1016/s0021-9258(18)68397-8 3379053

[B73] Nguyen A. D. Lee S. H. DeBose-Boyd R. A. (2009). Insig-mediated, sterol-accelerated degradation of the membrane domain of hamster 3-hydroxy-3-methylglutaryl-coenzyme A reductase in insect cells. J. Biol. Chem. 284 (39), 26778–26788. 10.1074/jbc.M109.032342 19638338 PMC2785366

[B74] Nguyen A. D. McDonald J. G. Bruick R. K. DeBose-Boyd R. A. (2007). Hypoxia stimulates degradation of 3-hydroxy-3-methylglutaryl-coenzyme A reductase through accumulation of lanosterol and hypoxia-inducible factor-mediated induction of insigs. J. Biol. Chem. 282 (37), 27436–27446. 10.1074/jbc.M704976200 17635920

[B75] Nohturfft A. Brown M. S. Goldstein J. L. (1998). Topology of SREBP cleavage-activating protein, a polytopic membrane protein with a sterol-sensing domain. J. Biol. Chem. 273 (27), 17243–17250. 10.1074/jbc.273.27.17243 9642295

[B76] Nunomura S. Endo K. Makishima M. Ra C. (2010). Oxysterol represses high-affinity IgE receptor-stimulated mast cell activation in Liver X receptor-dependent and -independent manners. FEBS Lett. 584 (6), 1143–1148. 10.1016/j.febslet.2010.02.006 20138879

[B77] Palade G. (1975). Intracellular aspects of the process of protein synthesis. Science 189 (4200), 347–358. 10.1126/science.1096303 1096303

[B78] Porter J. A. Young K. E. Beachy P. A. (1996). Cholesterol modification of hedgehog signaling proteins in animal development. Science 274 (5285), 255–259. 10.1126/science.274.5285.255 8824192

[B79] Radhakrishnan A. Goldstein J. L. McDonald J. G. Brown M. S. (2008). Switch-like control of SREBP-2 transport triggered by small changes in ER cholesterol: A delicate balance. Cell Metab. 8 (6), 512–521. 10.1016/j.cmet.2008.10.008 19041766 PMC2652870

[B80] Ravid T. Doolman R. AvneR R. Harats D. Roitelman J. (2000). The ubiquitin-proteasome pathway mediates the regulated degradation of mammalian 3-hydroxy-3-methylglutaryl-coenzyme A reductase. J. Biol. Chem. 275 (46), 35840–35847. 10.1074/jbc.M004793200 10964918

[B81] Roitelman J. Olender E. H. Bar-Nun S. Dunn W. A. Simoni R. D. (1992). Immunological evidence for eight spans in the membrane domain of 3-hydroxy-3-methylglutaryl coenzyme A reductase: Implications for enzyme degradation in the endoplasmic reticulum. J. Cell Biol. 117 (5), 959–973. 10.1083/jcb.117.5.959 1374417 PMC2289486

[B82] Roitelman J. Simoni R. D. (1992). Distinct sterol and nonsterol signals for the regulated degradation of 3-hydroxy-3-methylglutaryl-CoA reductase. J. Biol. Chem. 267 (35), 25264–25273. 10.1016/s0021-9258(19)74035-6 1460026

[B83] Sato R. Goldstein J. L. Brown M. S. (1993). Replacement of serine-871 of hamster 3-hydroxy-3-methylglutaryl-CoA reductase prevents phosphorylation by AMP-activated kinase and blocks inhibition of sterol synthesis induced by ATP depletion. Proc. Natl. Acad. Sci. U. S. A. 90 (20), 9261–9265. 10.1073/pnas.90.20.9261 8415689 PMC47547

[B84] Schumacher M. M. Elsabrouty R. Seemann J. Jo Y. DeBose-Boyd R. A. (2015). The prenyltransferase UBIAD1 is the target of geranylgeraniol in degradation of HMG CoA reductase. Elife 4. 10.7554/eLife.05560 PMC4374513 25742604

[B85] Schumacher M. M. Jun D. J. Jo Y. Seemann J. DeBose-Boyd R. A. (2016). Geranylgeranyl-regulated transport of the prenyltransferase UBIAD1 between membranes of the ER and Golgi. J. Lipid Res. 57 (7), 1286–1299. 10.1194/jlr.M068759 27121042 PMC4918857

[B86] Sever N. Song B. L. Yabe D. Goldstein J. L. Brown M. S. DeBose-Boyd R. A. (2003). Insig-dependent ubiquitination and degradation of mammalian 3-hydroxy-3-methylglutaryl-CoA reductase stimulated by sterols and geranylgeraniol. J. Biol. Chem. 278 (52), 52479–52490. 10.1074/jbc.M310053200 14563840

[B87] Sever N. Yang T. Brown M. S. Goldstein J. L. DeBose-Boyd R. A. (2003). Accelerated degradation of HMG CoA reductase mediated by binding of insig-1 to its sterol-sensing domain. Mol. Cell 11 (1), 25–33. 10.1016/s1097-2765(02)00822-5 12535518

[B88] Skalnik D. G. Narita H. Kent C. Simoni R. D. (1988). The membrane domain of 3-hydroxy-3-methylglutaryl-coenzyme A reductase confers endoplasmic reticulum localization and sterol-regulated degradation onto beta-galactosidase. J. Biol. Chem. 263 (14), 6836–6841. 10.1016/s0021-9258(18)68719-8 2834394

[B89] Smythe C. D. Greenall M. Kealey T. (1998). The activity of HMG-CoA reductase and acetyl-CoA carboxylase in human apocrine sweat glands, sebaceous glands, and hair follicles is regulated by phosphorylation and by exogenous cholesterol. J. Invest. Dermatol. 111 (1), 139–148. 10.1046/j.1523-1747.1998.00246.x 9665401

[B90] Song B. L. Javitt N. B. DeBose-Boyd R. A. (2005). Insig-mediated degradation of HMG CoA reductase stimulated by lanosterol, an intermediate in the synthesis of cholesterol. Cell Metab. 1 (3), 179–189. 10.1016/j.cmet.2005.01.001 16054061

[B91] Song B. L. Sever N. DeBose-Boyd R. A. (2005). Gp78, a membrane-anchored ubiquitin ligase, associates with Insig-1 and couples sterol-regulated ubiquitination to degradation of HMG CoA reductase. Mol. Cell 19 (6), 829–840. 10.1016/j.molcel.2005.08.009 16168377

[B92] Summons R. E. Bradley A. S. Jahnke L. L. Waldbauer J. R. (2006). Steroids, triterpenoids and molecular oxygen. Philos. Trans. R. Soc. Lond. B Biol. Sci. 361 (1470), 951–968. 10.1098/rstb.2006.1837 16754609 PMC1578733

[B93] Sun L. P. Li L. Goldstein J. L. Brown M. S. (2005). Insig required for sterol-mediated inhibition of Scap/SREBP binding to COPII proteins *in vitro* . J. Biol. Chem. 280 (28), 26483–26490. 10.1074/jbc.M504041200 15899885

[B94] Sun L. P. Seemann J. Goldstein J. L. Brown M. S. (2007). Sterol-regulated transport of SREBPs from endoplasmic reticulum to Golgi: Insig renders sorting signal in Scap inaccessible to COPII proteins. Proc. Natl. Acad. Sci. U. S. A. 104 (16), 6519–6526. 10.1073/pnas.0700907104 17428919 PMC1851663

[B95] Tao S. Y. Wang L. y. Yu X. f. Niu C. Pang C. j. (2012). Mutation in the UBIAD1 gene of a Chinese family with Schnyder crystal corneal dystrophy. Zhonghua Yi Xue Za Zhi 92 (45), 3215–3217. 23328470

[B96] Theofilopoulos S. Abreu de Oliveira W. A. Yang S. Yutuc E. Saeed A. Abdel-Khalik J. (2019). 24(S), 25-Epoxycholesterol and cholesterol 24S-hydroxylase (CYP46A1) overexpression promote midbrain dopaminergic neurogenesis *in vivo* . J. Biol. Chem. 294 (11), 4169–4176. 10.1074/jbc.RA118.005639 30655290 PMC6422085

[B97] Trinh M. N. Brown M. S. Seemann J. Vale G. McDonald J. G. Goldstein J. L. (2022). Interplay between Asters/GRAMD1s and phosphatidylserine in intermembrane transport of LDL cholesterol. Proc. Natl. Acad. Sci. U. S. A. 119 (2), e2120411119. 10.1073/pnas.2120411119 34992143 PMC8764668

[B98] Tsubaki M. Mashimo K. Takeda T. Kino T. Fujita A. Itoh T. (2016). Statins inhibited the MIP-1α expression via inhibition of Ras/ERK and Ras/Akt pathways in myeloma cells. Biomed. Pharmacother. 78, 23–29. 10.1016/j.biopha.2015.12.017 26898421

[B99] Von Gunten C. F. Sinensky M. (1989). Treatment of CHO-K1 cells with 25-hydroxycholesterol produces a more rapid loss of 3-hydroxy-3-methylglutaryl-coenzyme A reductase activity than can be accounted for by enzyme turnover. Biochim. Biophys. Acta 1001 (2), 218–224. 10.1016/0005-2760(89)90151-3 2917146

[B100] Weiss J. S. Kruth H. S. Kuivaniemi H. Tromp G. Karkera J. Mahurkar S. (2008). Genetic analysis of 14 families with Schnyder crystalline corneal dystrophy reveals clues to UBIAD1 protein function. Am. J. Med. Genet. A 146A (3), 271–283. 10.1002/ajmg.a.32201 18176953

[B101] Weiss J. S. Kruth H. S. Kuivaniemi H. Tromp G. White P. S. Winters R. S. (2007). Mutations in the UBIAD1 gene on chromosome short arm 1, region 36, cause Schnyder crystalline corneal dystrophy. Invest. Ophthalmol. Vis. Sci. 48 (11), 5007–5012. 10.1167/iovs.07-0845 17962451

[B102] Xie J. Li L. (2021). Functional study of SCCD pathogenic gene UBIAD1 (Review). Mol. Med. Rep. 24 (4), 706. 10.3892/mmr.2021.12345 34368857 PMC8365407

[B103] Yabe D. Brown M. S. Goldstein J. L. (2002). Insig-2, a second endoplasmic reticulum protein that binds SCAP and blocks export of sterol regulatory element-binding proteins. Proc. Natl. Acad. Sci. U. S. A. 99 (20), 12753–12758. 10.1073/pnas.162488899 12242332 PMC130532

[B104] Yan R. Cao P. Song W. Qian H. Du X. Coates H. W. (2021). A structure of human Scap bound to Insig-2 suggests how their interaction is regulated by sterols. Science 371: (6533), eabb2224. 10.1126/science.abb2224 33446483

[B105] Yang T. Espenshade P. J. Wright M. E. Yabe D. Gong Y. Aebersold R. (2002). Crucial step in cholesterol homeostasis: Sterols promote binding of SCAP to INSIG-1, a membrane protein that facilitates retention of SREBPs in ER. Cell 110 (4), 489–500. 10.1016/s0092-8674(02)00872-3 12202038

[B106] Yoshioka H. Coates H. W. Chua N. K. Hashimoto Y. Brown A. J. Ohgane K. (2020). A key mammalian cholesterol synthesis enzyme, squalene monooxygenase, is allosterically stabilized by its substrate. Proc. Natl. Acad. Sci. U. S. A. 117 (13), 7150–7158. 10.1073/pnas.1915923117 32170014 PMC7132291

[B107] Zelcer N. Sharpe L. J. Loregger A. Kristiana I. Cook E. C. L. Phan L. (2014). The E3 ubiquitin ligase MARCH6 degrades squalene monooxygenase and affects 3-hydroxy-3-methyl-glutaryl coenzyme A reductase and the cholesterol synthesis pathway. Mol. Cell. Biol. 34 (7), 1262–1270. 10.1128/MCB.01140-13 24449766 PMC3993563

[B108] Zerenturk E. J. Kristiana I. Gill S. Brown A. J. (2012). The endogenous regulator 24(S), 25-epoxycholesterol inhibits cholesterol synthesis at DHCR24 (Seladin-1). Biochim. Biophys. Acta 1821 (9), 1269–1277. 10.1016/j.bbalip.2011.11.009 22178193

[B109] Zhang Y. Motamed M. Seemann J. Brown M. S. Goldstein J. L. (2013). Point mutation in luminal loop 7 of Scap protein blocks interaction with loop 1 and abolishes movement to Golgi. J. Biol. Chem. 288 (20), 14059–14067. 10.1074/jbc.M113.469528 23564452 PMC3656263

[B110] Zhong C. Wang B. (2022). Regulation of cholesterol binding to the receptor patched1 by its interactions with the ligand sonic hedgehog (shh). Front. Mol. Biosci. 9, 831891. 10.3389/fmolb.2022.831891 35187087 PMC8847689

